# Supplementary material for: Mechanism of actin-dependent activation of nucleotidyl cyclase toxins from bacterial human pathogens
Source: Nat Commun. 2021 Nov 16;12:6628. doi: 10.1038/s41467-021-26889-2 (PMC8595890; doi:10.1038/s41467-021-26889-2)
Supplement: Supplementary file 1 — Supplementary Information [file 41467_2021_26889_MOESM1_ESM.pdf]

Mechanism of actin-dependent activation of nucleotidyl cyclase toxins from bacterial human pathogens

Alexander Belyy, Felipe Merino, Undine Mechold and Stefan Raunser

Supplementary Information

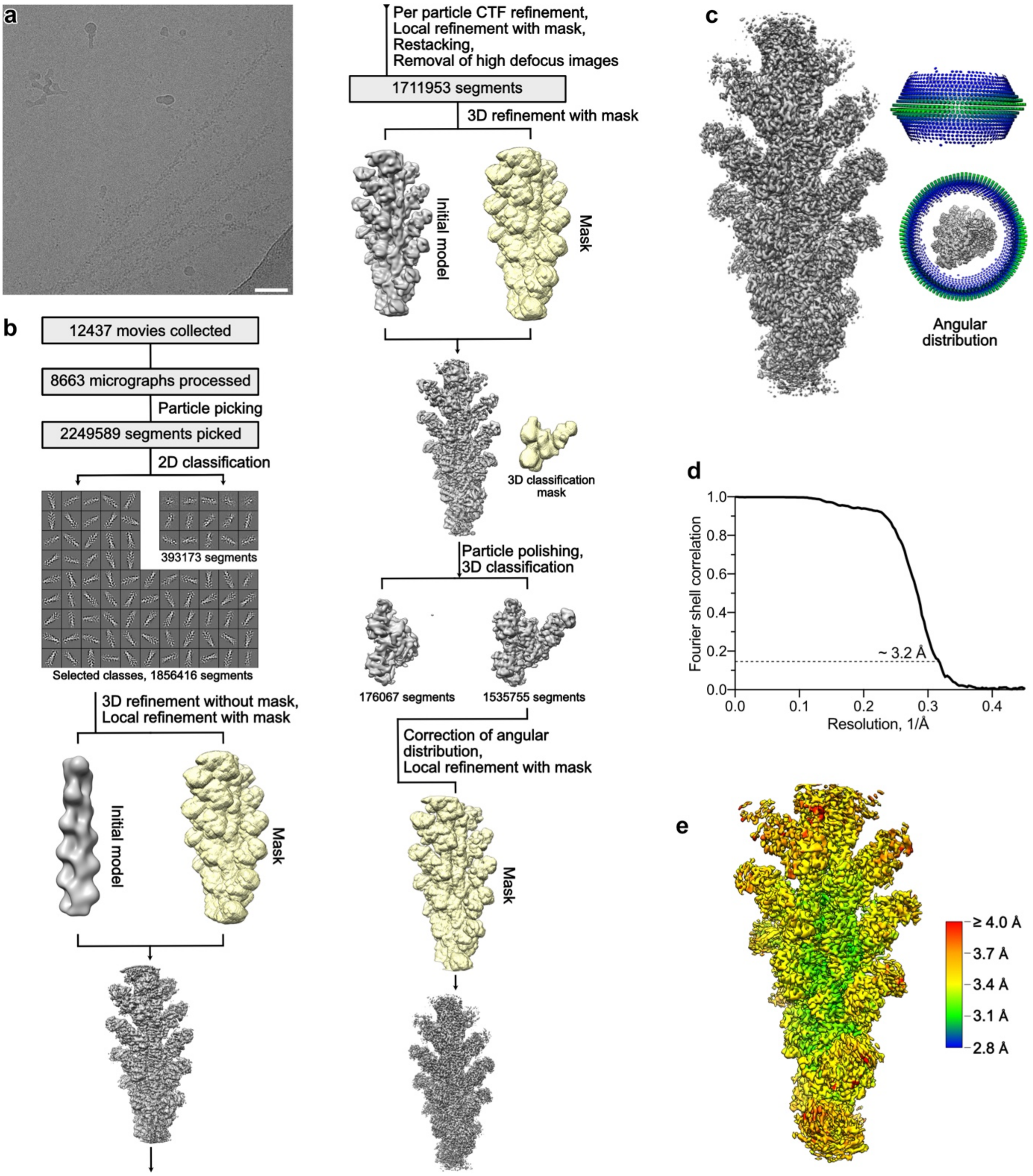

Supplementary Figure 1. Processing of the PaExoY-F-actin complex. a An example of the 8663 analyzed cryo-EM micrographs. Scale bar 50 nm. **b** Processing overview. **c** The final postprocessed map filtered according to the local resolution, and its angular distribution. **d** Fourier shell correlation curve. **e** Local resolution gradient of the reconstruction.

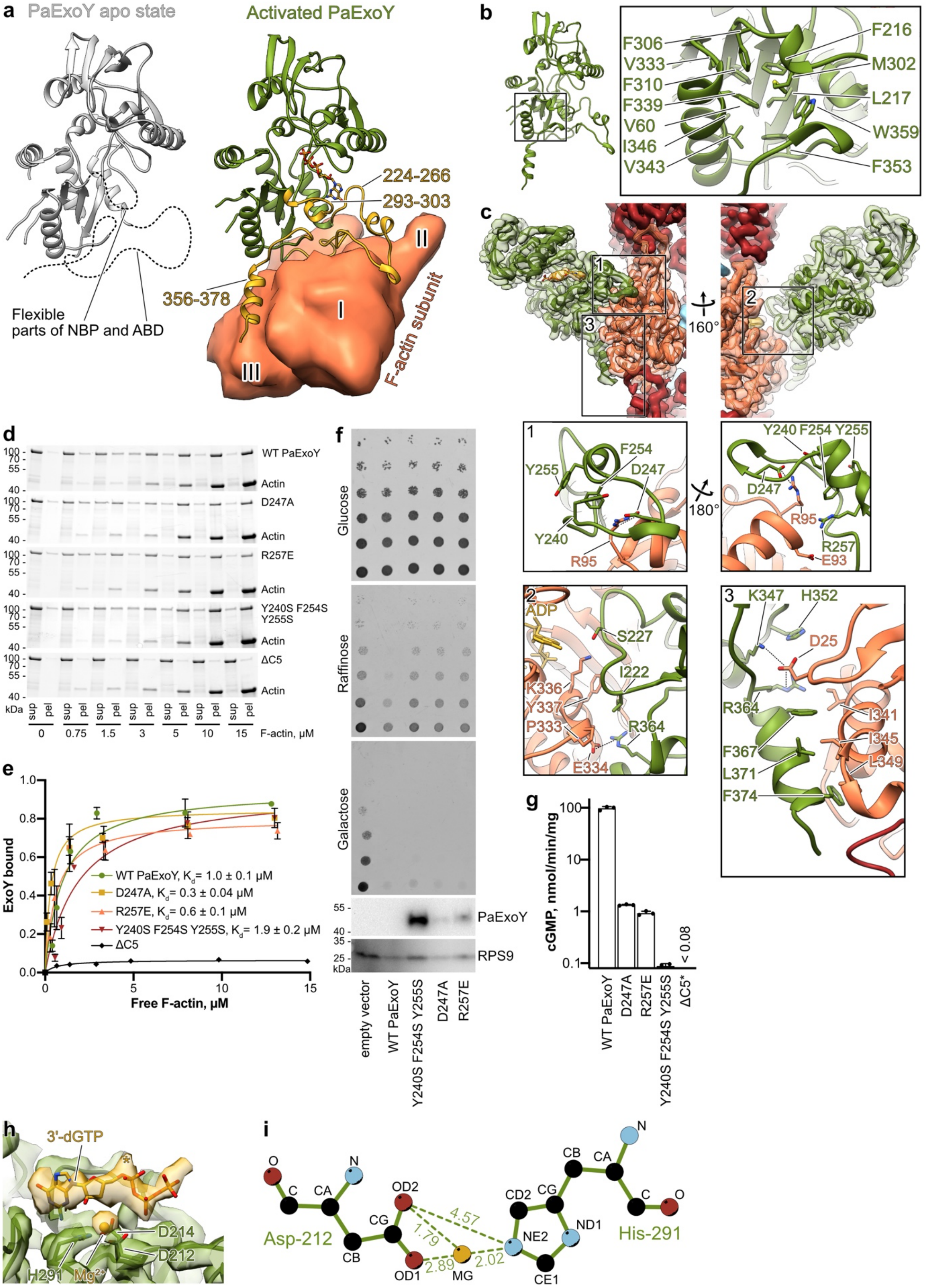

Supplementary Figure 2. PaExoY-F-actin complex. a Comparison of the PaExoY apo state structure[1] to its activated form. Dashed lines indicate the missing regions in the apo sate. These regions are fully resolved in the PaExoY-F-actin structure and are colored in yellow. Actin subdomains are marked by Roman numerals. **b** Hydrophobic core of PaExoY. **c** Overview of

the interaction sites. Close-up views: 1 – distal part of the sensor with the hydrophobic scaffold of Tyr-240, Phe-254 and Tyr-255 that position Asp-247 and Arg-257 towards Glu-93 and Arg-95 of actin; 2 – Ile-222 at the proximal part of the sensor creates contacts with Tyr-337 and Pro-333 of subdomain III of actin; 3 – the anchor starts with a loop where positively charged Lys-347 and Arg-364 lock Asp-25 of actin, and continues in a long helix that creates extensive hydrophobic contacts with the subdomain I of actin. **d** Cosedimentation of F-actin and 2.5 μM PaExoY analyzed by SDS-PAGE. The upper band corresponds to PaExoY variants, and the lower band corresponds to actin. Representative stain-free gels are shown, the experiment was performed 3 times independently. **e** The fractions of PaExoY that cosedimented with F-actin were quantified by densitometry and plotted against F-actin concentrations. **f** Growth phenotype assay with *S. cerevisiae* expressing PaExoY variants under a strong galactose promoter in the experimental conditions with background (Glucose), low (Raffinose) or high (Galactose) toxin expression. Analysis of protein expression was performed by Western blot of cells grown on galactose-containing media with anti-myc (PaExoY) and anti-ribosomal protein S9 (RPS9) antibodies. The western blot experiment was performed twice. **g** Activity of 30 ng of WT or 1 μg of PaExoY variants in the presence of 3 μM non-stabilized actin measured during 10 min of incubation. The data in panels e and g are presented as mean values, the error bars correspond to standard deviations of 3 independent experiments. ΔC5 is an PaExoY mutant with a deletion of 5 C-terminal amino acids. * - The measurement was performed previously[2]. **h** Atomic model of 3'-dGTP and Mg^{2+} in the catalytic center of PaExoY fit in the corresponding cryo-EM map. Asterisk symbol points to the position of the possible second metal ion. **i** Distances between Mg^{2+} and neighboring atoms. ABD – actin-binding domain, NBP – nucleotide-binding pocket. The uncropped gels and Western blots can be found in Supplementary Fig. 10.

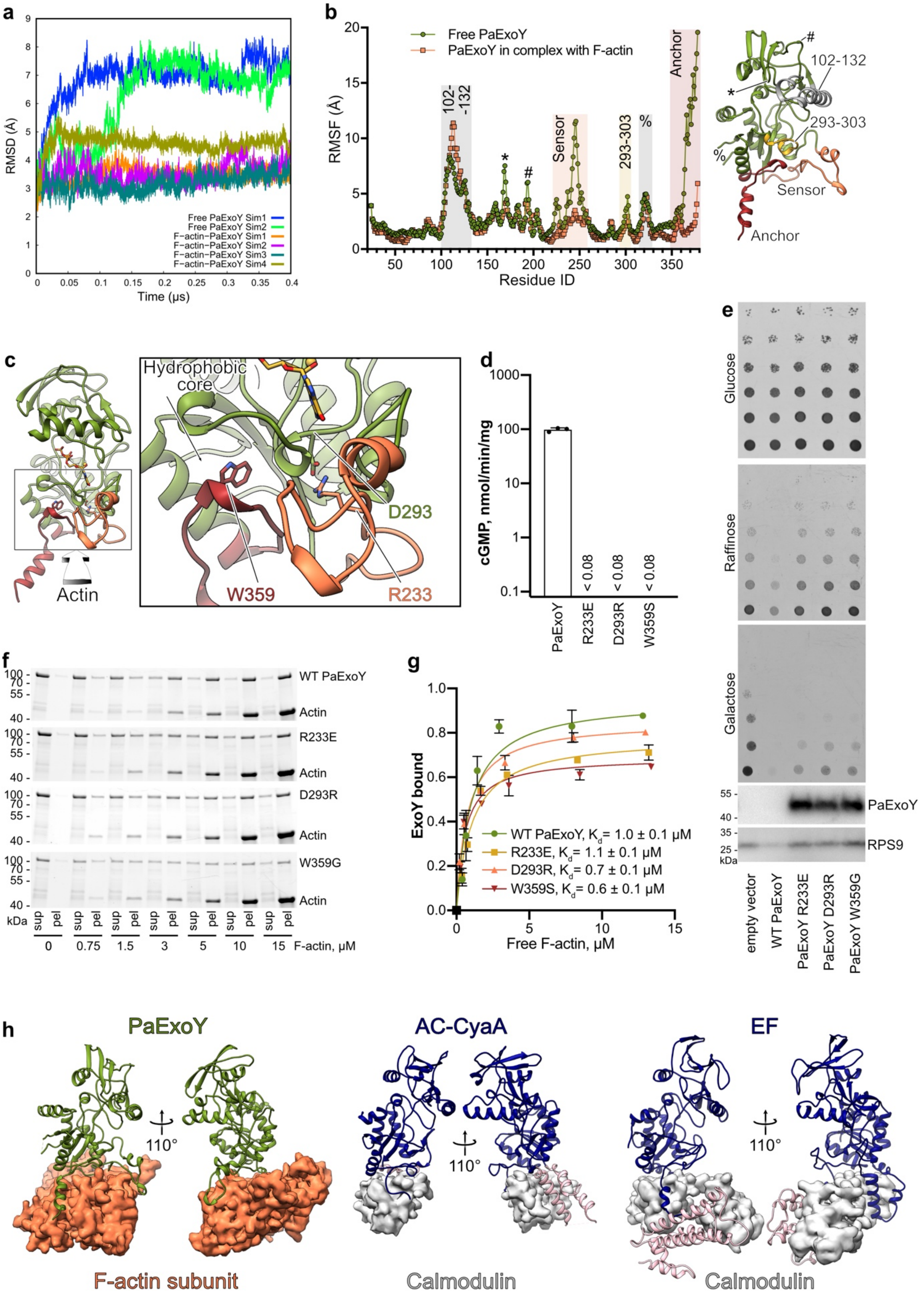

Supplementary Figure 3. Mechanism of activation of PaExoY. a Root mean square deviation (RMSD) of Cα atoms of PaExoY during the simulations. The initial structure is the reference for all simulations. For each system, all independent simulations are shown

separately. **b** Root mean square fluctuation (RMSF) of Cα atoms of PaExoY. For each system, we pooled simulations, superimposed all frames and calculated an overall RMSF. **c** Communication of activation signal through PaExoY to the nucleotide-binding pocket. **d** Activity of 30 ng of WT or 1 μg of PaExoY variants in the presence of 3 μM non-stabilized actin measured during 10 min of incubation. **e** Growth phenotype assay with yeast expressing PaExoY variants in the background (Glucose), low (Raffinose) or high (Galactose) level. Analysis of protein expression was performed by Western blot of cells grown on galactose-containing media with anti-myc (PaExoY) and anti-ribosomal protein S9 (RPS9) antibodies. The western blot experiment was performed twice. **f** Cosedimentation of F-actin and 2.5 μM PaExoY detected by SDS-PAGE. Representative stain-free gels are shown, the experiment was performed 3 times independently. **g** The fractions of PaExoY that cosedimented with F-actin were quantified by densitometry and plotted against F-actin concentrations. **h** Comparison of PaExoY and calmodulin-activated nucleotidyl cyclases (adenylyl cyclase from *B. pertussis*, AC-CyaA[3], PDB 1YRT; edema factor from *B. anthracis*, EF, PDB 1XFV [4]). The central interaction region of the AC-CyaA and the C-terminal interaction region in EF are in pink. Protective antigen binding domain of EF is hidden for the clarity of the figure. The data in panels d and g are presented as mean values, the error bars correspond to standard deviations of 3 independent experiments. The uncropped gels and Western blots can be found in Supplementary Fig. 10.

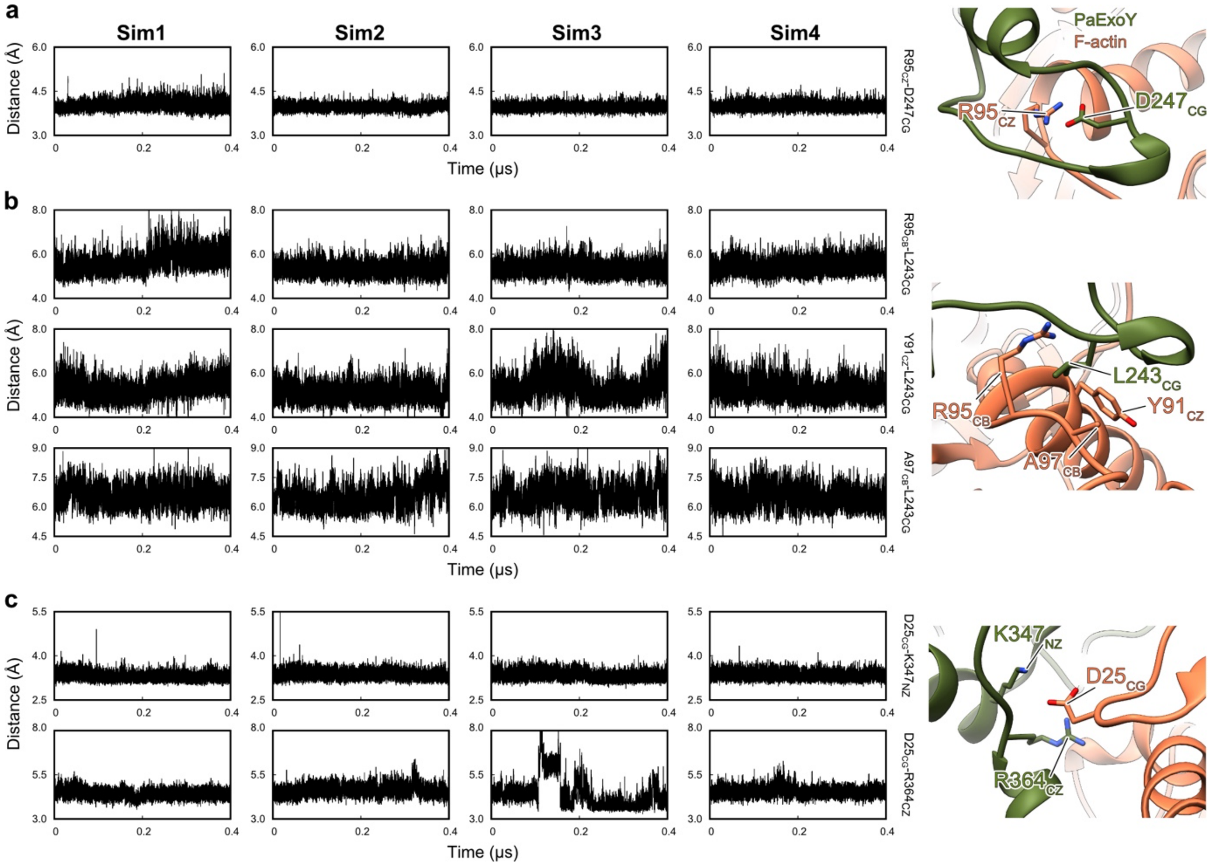

Supplementary Figure 4. Stability of different PaExoY-F-actin interactions during the MD simulations. For all interactions, the structure on the right shows the atoms used for the distance measurements. **a** Arg-95-Asp-247 of F-actin and PaExoY, respectively, **b** Interactions around PaExoY Leu-243, **c** Interactions of actin Asp-25.

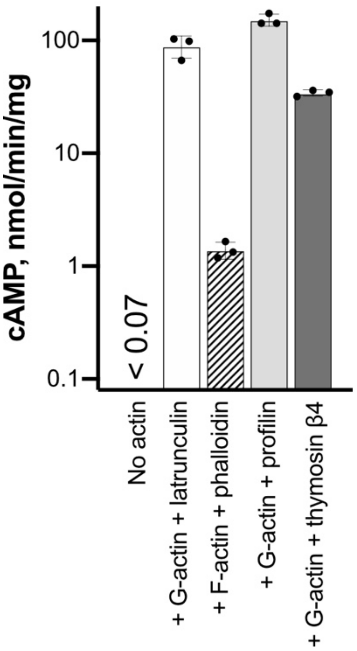

Supplementary Figure 5. Profilin-G-actin complex activates VvExoY. Activity of 4000 ng of VvExoY in the absence of actin; 10 ng of VvExoY in the presence of 2 μM of G-β-actin and 3 μM latrunculin A, 2 μM of G-β-actin and 3 μM profilin, or 2 μM of G-β-actin and 3 μM thymosin β4; 1000 ng of VvExoY in the presence of 2 μM of F-β-actin and 3 μM phalloidin during 10 min of incubation. The data are presented as mean values, the error bars correspond to standard deviations of 3 independent experiments.

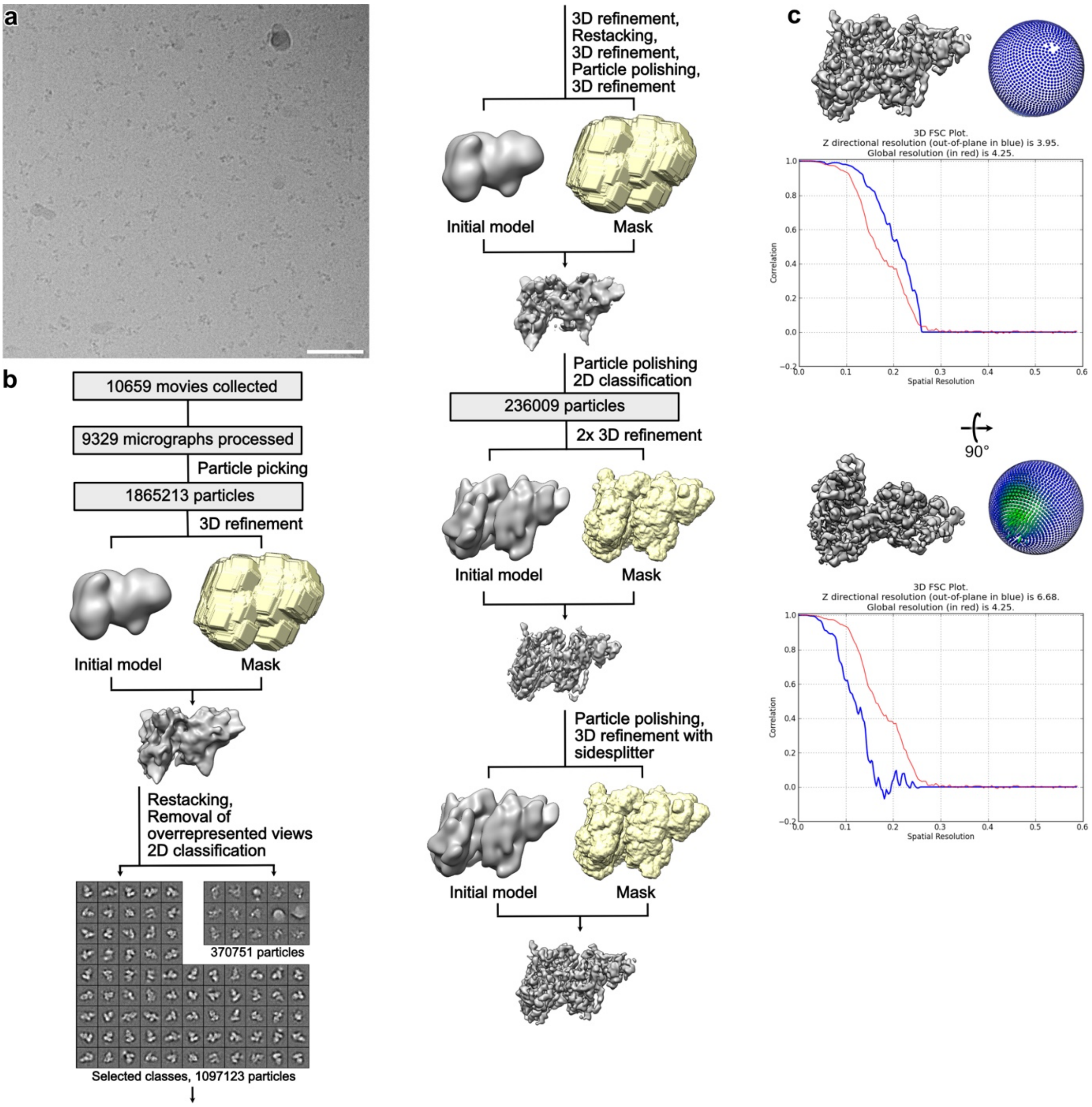

Supplementary Figure 6. Processing of the VnExoY-G-actin complex. a An example of the 9329 analyzed cryo-EM micrographs. Scale bar 50 nm. **b** Processing overview. **c** Map postprocessed by DeemEMhancer[5], its angular distribution, and FSC plots[6] in two orientations show strong preferred specimen orientation.

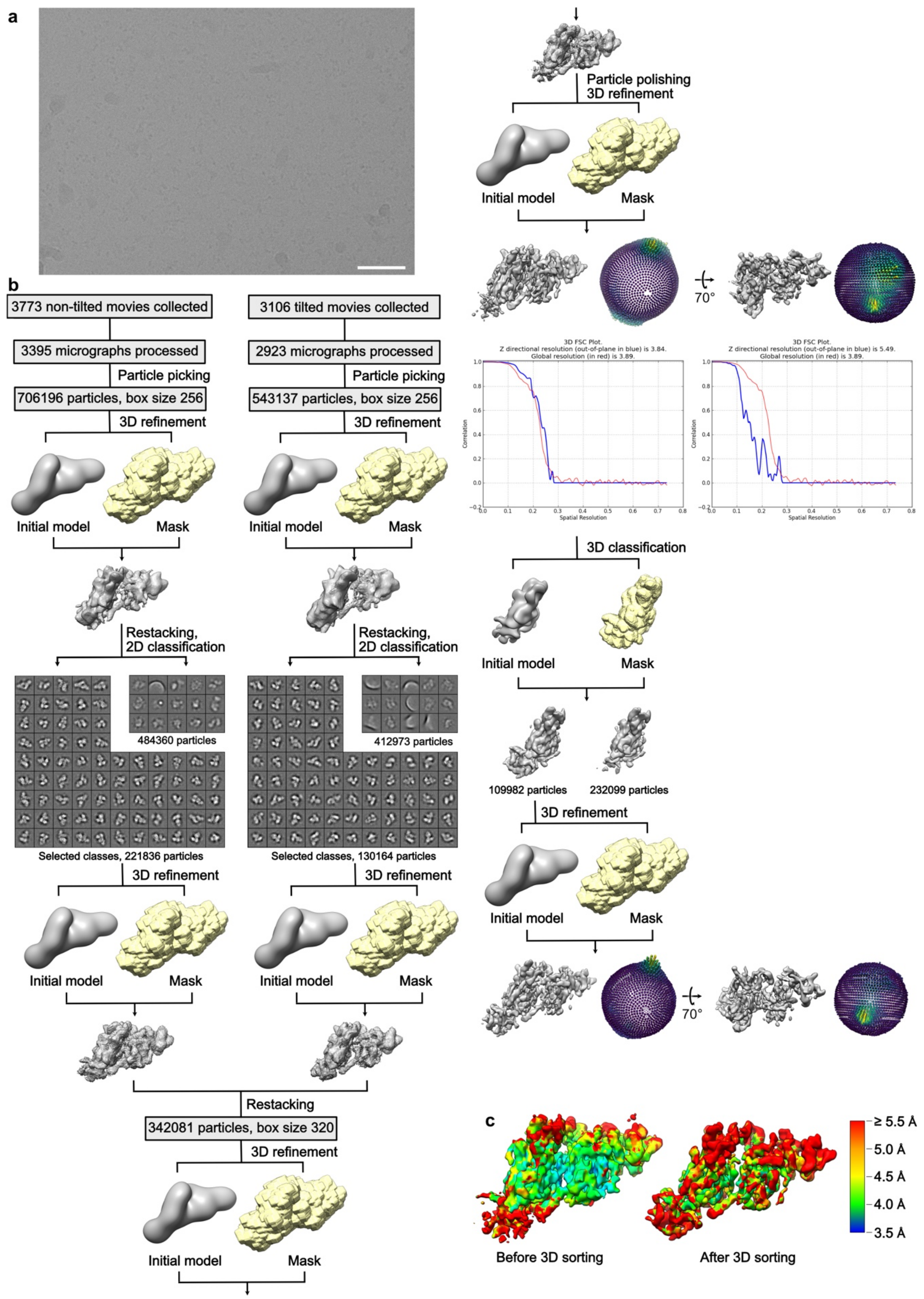

Supplementary Figure 7. Processing of the VvExoY-G-β-actin complex. a An example of the 6318 analyzed cryo-EM micrographs. Scale bar 50 nm. **b** Processing overview with postprocessed maps, their angular distribution, and FSC plots[6]. **c** Local resolution gradient of the reconstructions before and after 3D sorting. A combination of these maps was used for Fig 3A.

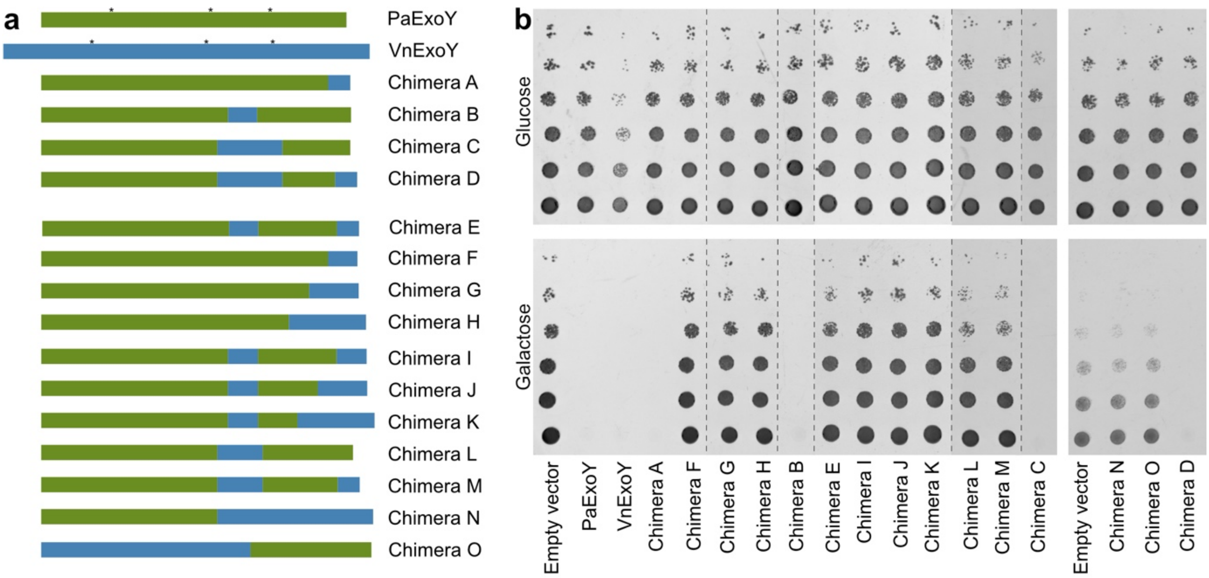

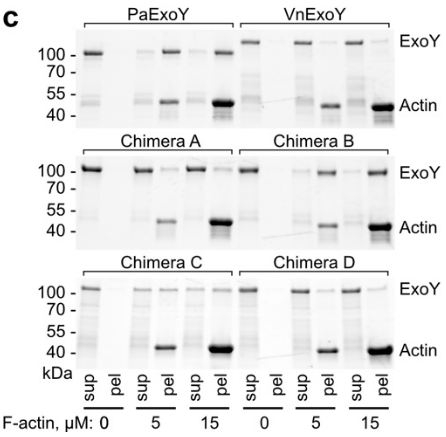

d

	Activator	Substrate
PaExoY	F > G-actin	GTP > ATP
VnExoY	G >> F	ATP only
Chimera A	G = F	GTP > ATP
Chimera B	F > G	ATP > GTP
Chimera C	G > F	ATP only
Chimera D	G > F	ATP only

Supplementary Figure 8. All chimera proteins created and tested in this study. a A schematic representation of the created chimera proteins. **b** Yeast viability upon endogenous chimera expression on the background (Glucose) or high (Galactose) level. The panel is composed of several drop-test images as indicated by dashed lines. Amino acid sequences of the chimera proteins are available in Supplementary Table 3. **c** Cosedimentation of F-actin and 2.5 μM chimera proteins detected by SDS-PAGE. Representative stain-free gels are shown, the experiment was performed 3 times independently. The uncropped gels can be found in Supplementary Fig. 9. **d** Overview of activator preference and substrate specificity of ExoY variants.

a

F-actin activated:

```
Pa   212 DYDLFLVAPSIEAHGSGGLDARRNTAVRYTPLGAK-DPLSEDG--FYGRE-------DMARGNITPRTRQLVDALNDCLGRGEHREMFHH 291
As       DYDMFLVAPSIEEYGQGGSDARTNTAVKYQPLPN--DPHTMAS--FYGRD-------DVTKGNISPRIRDLVDTLNDTLGRGEHRDMFHH
         ***.********  * ** *** ****.* **    **        ****.        *  .***.** * *** *** *******.****

Pa   347 KGNGYHFTAHPDWNVPLRP---------SFQEALDFFQRKV  378
As       KGNGYHFNAHPDWQIPQRL---------SFQEAFAFFNQKV
         ******* *****..* *          *****. **. **
```

G-actin activated:

```
Vv   213 DYDLLTVMYSYSDLGPQDKLKQPLTWEQWKESVT-YEELTPKYKELYNSEVLYNKKDGASLGVVSDRLKALKDVINTSLGRTDGLEMVHH 301
Vn       DYDLLTVMYTYGDLGPQDKVKQPLTWEQWKESVT-YEDLSPKYKARYDNQALYEKQDGASLGMVSDRLKELKDVINTSLGRTDGLEMVHH
Va       DYDLLTVMYSYADLGPQDKVKQPLTWEQWKESVT-YEDLTPKYQELYDSEVLYNKKDGASLGVVSDRLKTLKDVINTSLGRTDGLEMVHH
Pm       DYDLFTVMYSYNDLSERSTVRKAIPWEEWRSSVN-YDELSPTYQEYYNNKDLYDRYEGEQLGIISQHVKNIKNKLNRLLQREKGKEMIHH
Vc       DYDLFSVMTHISDYGAPDMFKMPEPWADWKKSIKDPEKLDPKIKELFENEDLYNKVNNMHLGIISDRVIGLKDKINDRLGRNNGMELVHH
Vs       DYDLFSVMAHISDYGVSDLVKVPLPHAKWKATIENPDKLSPNLKELYDNEDLYNKVHNMHLGAISERVNSLKDKVNNRLGRGKGMELIHH
Vo       DYDLFTVMYSYAELGPQDKVKQPLTWEQWKESVT-YEDLTPKYKELYDSEVLYNKKDGASLGVVSDRLKTLKDVINTSLGRTDGLEMVHH
Pru      DYDLFSIIFPISELEHYAKVTEMPSWAEWKSGIN-YDELTVYQKALYENEAEYNKQEGRDNGITNSKIKEIKNKLNRRLGRTDGMELIHH
Pre      DYDLFSVIYPVSELEHYVKVSEMPSWQEWKASVN-YDELTNEQKKLYHNEAEYNKREGKDNGITNAKIKEINQELNKKLERPIGLELVHH
         ****..*.. . ..      .          **  .   . *        ..      *         *.     .   .   .*  * *  *.*..**

Vv   367 INVSYRASLNDKWNVGLDDPLFTPKSKLSHDFLNAKEEVIKKLSGEVETNVRTTQLLTDNEGL  425
Vn       INVSLGNYNFNWGGDGRDLGAYLGDNNNFWGGRGDDVYYATGTSNLFTGGEGQDMGVLMGREN
Va       INVSYRASLNDKWNAGLDDQLFTPKRKLSHEFLVARDKIAKMLSNESSDLGNDSELEGIAERR
Pm       INNGFISPLNEKWNEGIEGNYFSRRRKSSENYLNNRELLEKIFSDKNKYKSPLNKINNEYSDY
Vc       TNVHFTGALNTKWSENYAQL--ARKNRLSHAYLNAREQVDQLLFGKIDAAKIEFYRPKPFDET
Vs       TNVQFTGPLNSLWSKEYGQL--ARKNRLSHAYLNAREQVEALLTQGIDVAKLKVYIPGVFDEM
Vo       INVSYRASLNDKWNAGLDDPLFTPKRKLSHEFLNVRDELANKLKEKAVDFEKEVQLDRLGDHQ
Pru      INQGYRAPLNKIWNEGDNGQYFDPKRKISESFIEGRIEIARKKSLANELNDGFGYEGIRKSNR
Pre      INEGYRAPLNANWREGEYAEYFNPKRKISDSYINASREIQRKKSIPIAVRNFIEGKETQSGYI
          *   .                ..   *
```

b

```
Pa   212 DYDLFLVAPSIEAHGSGGLDARRNTAVRYTPLGAK-DPLSEDG--FYGRE------------------DMARGNITPRTRQLVDALNDCLGRGEH-REMFHH 291
Vv   213 DYDLLTVMYSYSDLGPQDKLKQPLTWEQWKESVT-YEELTPKYKELYNSEVL-----------YNKKDGASLGVVSDRLKALKDVINTSLGRTDG-LEMVHH 301
Bps      DYDLFLIGPHITDLGPQDNLPVPDVSHAVFKARLDKYKNG------IP--S-ALRSAYEQSGEFYRKEDEEIGNASLRVRNMIPLINTALVG-DG-EAVVHH
Mt       DYDLMIVAPRAEDFGSQDIPHNPD-VSADAFAEKRPY-LL------R--RG-STKEIIRRELL--EREDPHFGNNTPRITALILQINAVLFRGEG-REVVHH
Ya       DYDLLMVAPHISDLGSQDNLPVPDVAHHVFRRRIDKY-SK------WPDAP-EIRQDYDKPASFYRKEDGEIGNASERVRQMIPYLNEAVVG-DG-EKVIHH
Pf       DYDLHMVAPHISDLGPQDRLPVPDIAHSVFTTRVDHY-RQQQPDPKAFLVPEALRADYESAEHFYQKENPDLGNATPRIEQMIRLINDRLVTPPSEERVVHH
Prs      DYDLLLIGPHMRDFGHQDMLPVPDVAHSVYRQRVEKY-QR------LPTDA-NLSQAYRDENHFYQNEDSEIGNASPRVREMISLINRALVG-EA-EKVVHH
CHd      DYDLFMIAPSVTDFGFQDILPVKDVSHSIFKRKVKQYIKD------LS--E-PLKELYSDPDTFYAPEDEEIGNASLRIRDMISDINQHLVG-NG-EKVVHH
Cd       DYDLMLIAPHVRDLGREDNLQVPDVSHQVFRQRLESY-SK------LPLHP-ELRRDYEDAASFYRRSDEEMGNSSQRVRHMVGVINQALVG-KG-EPVVHH
Cv       DYDLLVIGPHLSDVGPQDNLPVPDVAHSVFRDRIDHY-RG------IPANV-GLLDAYLNPKSFYRREDAEIGNASARIRDMIPVINQALVG-DG-ERVVHH
Als      DYDLLLVAPNLSDYGAGDLAKGDP-SQSVLESHLR---------------------NFNAPKET-TQPKAPQGFISSRVKELGDDINARLDRE-L-NKLIHH
Ei       DYDLFIASPHISDFGSQDNLLIPDVAHKVFQHRVNSYHKE----LT--T-NHELMQDYSNADSFYKNEDPDIGNASARVRILIDLINATLVG-DG-ERLVHH
         ****   .        *.          .                                        *    *   .   **  .        ..**

Pa   347 KGNGYHFTAHPDWNVPLRP---------SFQEALDFFQRKV---------------------- 378
Vv   367 INVSYRASLNDKWNVGLDDPLFTPKSKLSHDFLNAKEEVIKKLSGEVETNVRTTQLLTDNEGL 425
Bps      KNAGYHVQINPMWEKEVASV--QRESFLTARGYFSAN-TRNIHPLR-----------------
Mt       ECHGFMVPHNPLWSDEIPSL--RRE---SFEATRKLFNTL-----------------------
Ya       KRHGYHMVMNPLWEPDLLQI--RNP---HFEEMRRELILKL----------------------
Pf       KDSGYHVPLNPLWESEVVSI--KRTGFTHAQARLASAL----RQ-------------------
Prs      KAEGYHIKTNPLWEKELPTI--RRPSFEYAKRRLST-V--SLNSK-------------I----
CHd      KDAGYYVPSNPLWEKDITSI--RSS---SFKAAKKVLIGHCAKIIKPK---------------
Cd       KNAGYHVPLNPLWEKEVLRA--RSP---RFDDARARIEAAM---L-ARH--------------
Cv       KDSGYHLPLNPLWEKEVSGV--RRE---SFVDARRRLGG------------------------
Als      KDQAFFVPVHNEWGQDVQNI--KRG---SFQESLDYFQKV-----------------------
Ei       RSSGYYIKINPLWEKELGKI--DSQ---RFKDAKVKLDP-L---TKPS---------------
          . .    *.
```

```
Pa   347 DYDLFLVAPSIEAHGSGGLDARRNTAVRYTPLGAK-DPLSEDG--FYGRE----------------------------------------DMARGNITPRTRQLVDALNDCLGRGEH---REMFHH 291
Vv   367 DYDLLTVMYSYSDLGPQDKLKQPLTWEQWKESVT-YEELTPKYKELYNSEVL-----------YNKKD----------------------GASLGVVSDRLKALKDVINTSLGRTDG---LEMVHH 301
Ob       DYDLMAVCPRWSDY--GSTSSAQIAK-PGLVMNGM-APRLGQVFERGTNLDKVLTMESSTGRPR--PQGWQAP--------AGF-GNEEHYDMGNVTPRILRCINLLNGRMVNGNGA--FRRVHH
Rs       DYDLMSVCPTWDSY--GGTSNAVISK-PGIRFSGK-KGLQLGQQFDVGARMDKVLDMRSNTGAR-PKLGNTKVTFQGMTKRDGG-KLAEHGDMGNITPRILRCINMLNAAMGATGGKSAGRRVHH
Mv       DYDLFAIWPFVGSY--DASAYGLDHR-PLGTVKGSVT-Q-AE---------------R---------DNVENLE--------RHFTEGGQGTKLGNITPRIYLICQLVNSIIAK------QQVLWH
En       DVDPLLEAFPLSDLDLGGKDKLPLPLISDVIVRKQVETYKNRLKSQFMEDTITAEQFVMKLSHYHDLEKELLKDFFTGITADGEHFEKEDPEMGNVTQRTRNMVSHYKRELQT--Q---EDVVHH
         *.* .    .                                                                                    * .. *        .          . *

Pa   347 KGNGYHFTAHPDWNVPLRP---------SFQEALDFFQRKV----------------------  378
Vv   367 INVSYRASLNDKWNVGLDDPLFTPKSKLSHDFLNAKEEVIKKLSGEVETNVRTTQLLTDNEGL  425
Ob       QDAGYFLPRNAAWGMSIRD-A-MRA-------V------------------------------
Rs       NEAGYYVPRNWTWGMSVRD-N-AAD---MQRYF------------------------------
Mv       DEAGIKVTLSNAWAQEPTA-QKPNRLGASYAGYVPADGVRIIVPDWYNA--------------
En       MEAGYEPEKNLLWGKTRARRPSFNQALHHLEGKTLSQQQKDKQLGEEIEKYWDSGISSESNEL
            .       *
```

Supplementary Figure 9. Alignment of PaExoY and VvExoY with known ExoY-like nucleotidyl cyclases. a Eleven ExoY proteins can be clearly classified into F-actin-activated (above) and G-actin-activated (below) based on their similarity in the ABD with PaExoY and VvExoY. **b** Fourteen other ExoY-like proteins differ in the activator-binding domain from the corresponding parts in PaExoY and VvExoY and from calmodulin-binding nucleotidyl

cyclases, suggesting the existence of at least one additional subgroup of bacterial nucleotidyl cyclases. Amino acids that are involved in direct contacts with actin or that organize a structural scaffold for actin-binding regions are in **bold**. Pa – *P. aeruginosa* ExoY WP_003115517, As - *Aeromonas salmonicida* ALK43954.1, Vv – *V. vulnificus* ExoY WP_039507922, Vn – *V. nigripulchritudo* WP_013610353.1, Va – *V. anguillarum* YP_004566017.1, Pm – *Proteus mirabilis* WP_020945177.1, Vc – *V. cholerae* AAW80256.1, Vs – *V. scophthalmi* 005594994.1, Vo – *V. ordalii* WP_010319615.1, Pru – *Providencia rustigianii* WP_006813565.1, Pre – *Providencia rettgeri* WP_004909604.1, Bps – *Burkholderia pseudomallei* KGC96437.1, Mt – *Mycobacterium tuberculosis* SGC81937.1, Ya – *Yersinia aldovae* WP_004701884.1, Pf – *P. fluorescens* WP_012722909.1, Prs - *Providencia* stuartii 014658369.1, CHd - *Candidatus Hamiltonella defensa* WP_015873608.1, Cd – *Cedecea davisae* WP_016538267.1, Cv – *Chromobacterium vaccinii* WP_083340618.1, Als – Algicola sagamiensis WP_083938267.1, Ei – *Edwardsiella ictaluri* YP_002932933.1, Ob – *Oxalobacteraceae* bacterium WP_020701664.1, Rs – *Rhodoferax saidenbachensis* WP_029708193.1, Mv – *Methylomerinum vadi* WP_031434121.1, En – *Endozoicomonas numazuensis* WP_034835431.1. The alignment was performed using T-Coffee[7] and adjusted manually.

Figure S2D and S3F

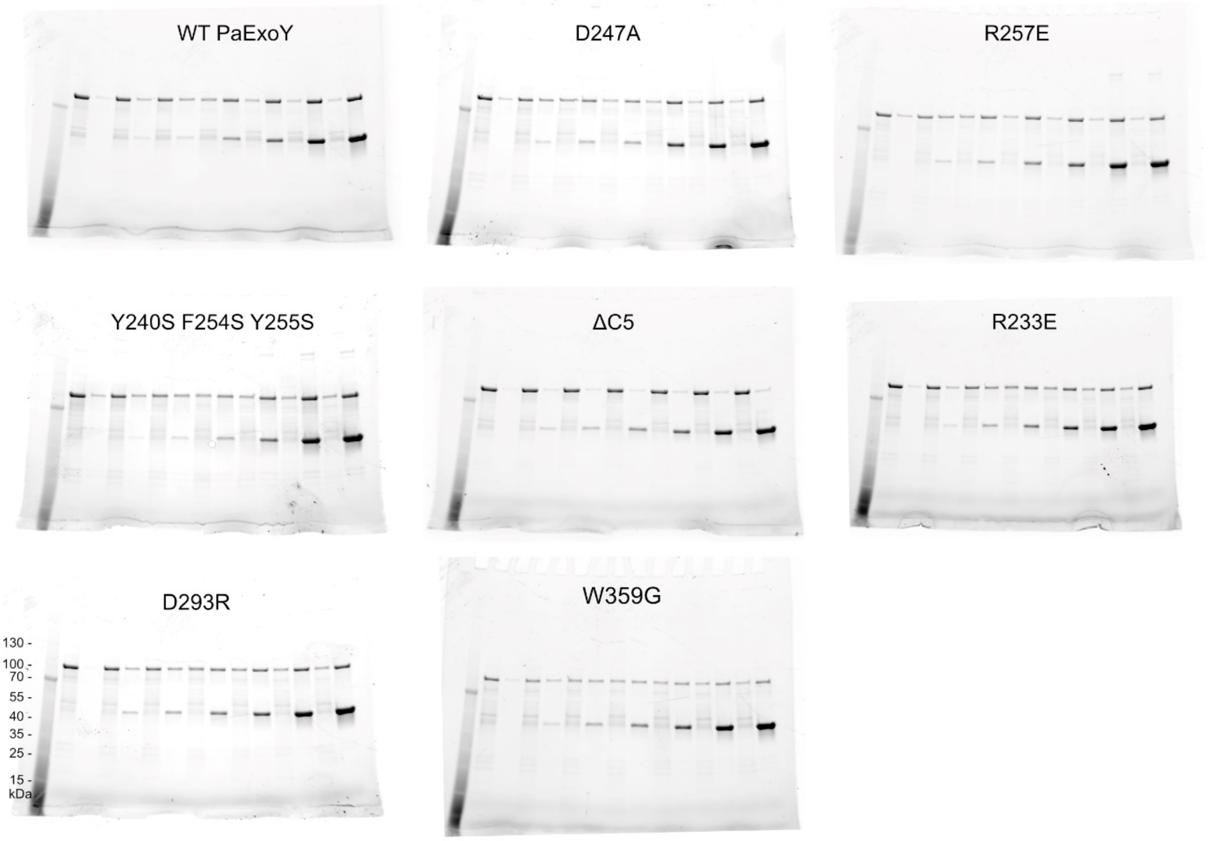

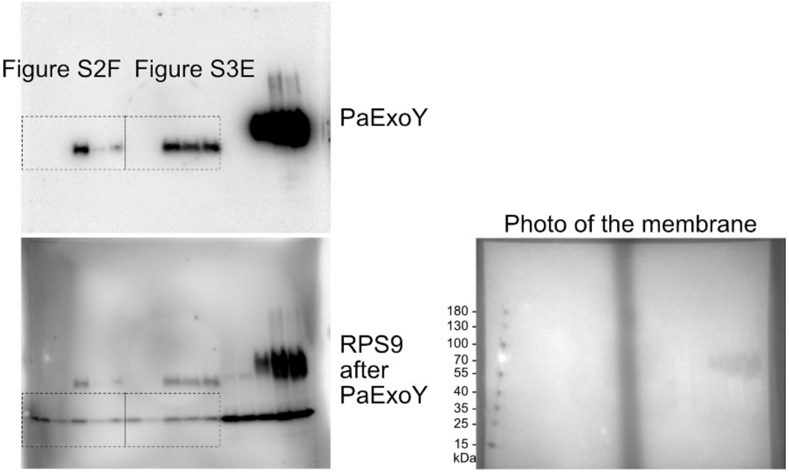

Figure S8C

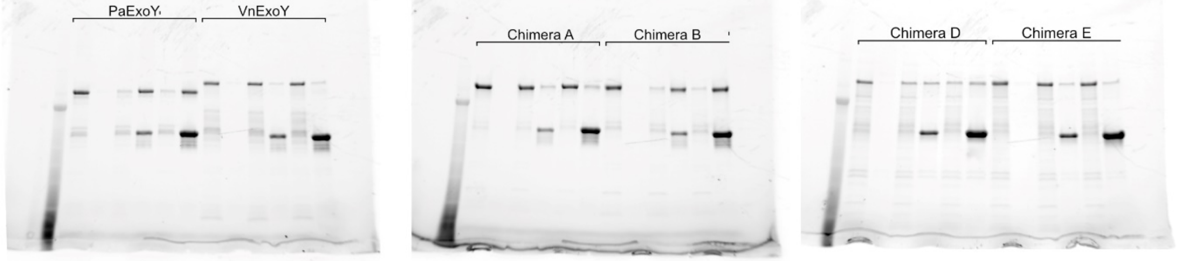

Supplementary Figure 10. Uncropped gels and Western blots.

Supplementary Table 1. Cryo-EM data collection, refinement, and validation statistics

Project	PaExoY-F-α-rabbit actin	VnExoY-G-α-rabbit actin	VvExoY-G-β-human actin-profilin
Microscope	Titan Krios	Titan Krios	Titan Krios
Voltage (kV)	300	300	300
Defocus range (µm)	-0.4 to -3	-1 to -2.5	-1.2 to -2.5
Camera	Falcon III (Linear mode)	Gatan K2 (Counting mode)	Gatan K3 (Superresolution mode)
Pixel size (Å)	1.1	0.85	0.34, 0.68 binned
Total electron dose (e/Å^2)	93	80	60
Exposure time (s)	1.5	8	2
Frames per movie	40	64	60
Number of movies	8,663 (12,437)	9,329 (10,659)	6,318 (6,880)
**3D Refinement**			
Number of particles	1,535,755 (2,249,589)	236,009 (1,865,213)	342,081 (1,299,405)
Final resolution (Å)	3.2	4.2	3.9
Helical rise (Å)	27	-	-
Helical twist (°)	-166.9	-	-
**Atomic model statistics**			
Non-hydrogen atoms	28580	-	7073
Molprobity score	1.89	-	1.98
Clashscore	9.1	-	12.09
EMRinger score	3.22	-	1.49
Bond RMSD (Å)	0.021	-	0.005
Angle RMSD (°)	1.332	-	1.024
Poor rotamers (%)	0.17	-	0
Favored rotamers (%)	94.80	-	92.95
Ramachandran favored (%)	93.99	-	94.25
Ramachandran allowed (%)	6.01	-	5.75
Ramachandran outliers (%)	0	-	0
Missing regions in the atomic model	PaExoY: 1-23, 318-325		VvExoY: 1-18 Actin: 41-47

Supplementary Table 2. List of primers, strains and plasmids used in this study.

**Bacterial and yeast strains**	**Description**	**Reference**
E. coli DH5α	F^- Φ80*lac*ZΔM15 Δ(*lac*ZYA-*arg*F) U169 *rec*A1 *end*A1 *hsd*R17(r_k^-, m_k^+) *pho*A *sup*E44 *thi*-1 *gyr*A96 *rel*A1 λ^-	Invitrogen
E. coli BL21 DE3 CodonPlus RIPL	F^- *ompT hsdS*(r_B^- m_B^-) *dcm*$^+$ Tet^r *gal*λ(DE3) *endA* Hte [*argU proL* Cam^r] [*argU ileY leuW* Strep/$Spec^r$]	Agilent
S. cerevisiae MH272-3fα	"Wild-type" strain, *ura3, leu2, his3, trp1, ade2*	[8]
S. cerevisiae Y395	*S. cerevisiae* MH272-3fα + Vector[ADE2] (2473)	[2]
S. cerevisiae Y410	*S. cerevisiae* MH272-3fα + PaExoY[ADE2] (p1593)	[2]
S. cerevisiae Y655	*S. cerevisiae* MH272-3fα + VnExoY[ADE2] (p1648)	[9]
S. cerevisiae Y642	*S. cerevisiae* MH272-3fα + PaExoY_Y240S_F254S_Y255S[ADE2] (pB636)	This study
S. cerevisiae Y595	*S. cerevisiae* MH272-3fα + PaExoY_D247A[ADE2] (pB568)	This study
S. cerevisiae Y593	*S. cerevisiae* MH272-3fα + PaExoY_R257E[ADE2] (pB566)	This study
S. cerevisiae Y532	*S. cerevisiae* MH272-3fα + PaExoY_R233E[ADE2] (pB490)	This study
S. cerevisiae Y603	*S. cerevisiae* MH272-3fα + PaExoY_D293R[ADE2] (pB582)	This study
S. cerevisiae Y608	*S. cerevisiae* MH272-3fα + PaExoY_W359S[ADE2] (pB587)	This study
S. cerevisiae Y722	*S. cerevisiae* MH272-3fα + VnExoY_W363S[ADE2](pB737)	This study
S. cerevisiae Y728	*S. cerevisiae* MH272-3fα + VnExoY_W222S_W225S_Y248S[ADE2] (pB743)	This study
S. cerevisiae Y732	*S. cerevisiae* MH272-3fα + VnExoY_ΔC27[ADE2] (pB747)	This study
S. cerevisiae Y656	*S. cerevisiae* MH272-3fα + ExoY_chimeraA[ADE2] (pB672)	This study
S. cerevisiae Y664	*S. cerevisiae* MH272-3fα + ExoY_chimeraB[ADE2] (pB678)	This study
S. cerevisiae Y665	*S. cerevisiae* MH272-3fα + ExoY_chimeraC[ADE2] (pB681)	This study
S. cerevisiae Y747	*S. cerevisiae* MH272-3fα + ExoY_chimeraD[ADE2] (pB756)	This study
S. cerevisiae Y717	*S. cerevisiae* MH272-3fα + ExoY_chimeraE[ADE2] (pB731)	This study
S. cerevisiae Y657	*S. cerevisiae* MH272-3fα + ExoY_chimeraF[ADE2] (pB673)	This study
S. cerevisiae Y660	*S. cerevisiae* MH272-3fα + ExoY_chimeraG[ADE2] (pB679)	This study
S. cerevisiae Y661	*S. cerevisiae* MH272-3fα + ExoY_chimeraH[ADE2] (pB680)	This study
S. cerevisiae Y666	*S. cerevisiae* MH272-3fα + ExoY_chimeraI[ADE2] (pB682)	This study
S. cerevisiae Y667	*S. cerevisiae* MH272-3fα + ExoY_chimeraJ[ADE2] (pB683)	This study
S. cerevisiae Y668	*S. cerevisiae* MH272-3fα + ExoY_chimeraL[ADE2] (pB684)	This study
S. cerevisiae Y697	*S. cerevisiae* MH272-3fα + ExoY_chimeraL[ADE2] (pB717)	This study
S. cerevisiae Y698	*S. cerevisiae* MH272-3fα + ExoY_chimeraM[ADE2] (pB719)	This study
S. cerevisiae Y715	*S. cerevisiae* MH272-3fα + ExoY_chimeraN[ADE2] (pB729)	This study
S. cerevisiae Y716	*S. cerevisiae* MH272-3fα + ExoY_chimeraO[ADE2] (pB730)	This study
**Plasmids for experiments in *S. cerevisiae***		
2473 YEpGal555	*E. coli*/*S. cerevisiae* shuttle vector [ADE2] with Gal1 promoter	[10]
p1593 YEpGal555 PaExoY	WT PaExoY with N-terminal myc-tag in YEpGal555 vector	[2]
p1648 YEpGal555 VnExoY	WT VnExoY with N-terminal myc-tag in YEpGal555 vector	[9]
pB576 YEpGal555 PaExoY_Y240S	The Y240S mutation was generated by two-step overlap PCR using oligonucleotides cagcgaagcgatgatttttg, caggttgtctaactccttcc,	This study

	ccgcggtcagatccaccccccctcgg and ccgaggggggtggatctgaccgcgg, and p1593 as a matrix. The PCR product was digested with XhoI and KpnI and ligated into digested 2473 YEpGal555 vector.	
pB636 YEpGal555 PaExoY_Y240S_F254S_Y255S	The F254S and Y255S mutations were generated by two-step overlap PCR using oligonucleotides cagcgaagcgatgatttttg, caggttgtctaactccttcc, ctgagcgaggacggatccagtggcagggaggatatg and catatcctccctgccactggatccgtcctcgctcag, and pB576 as a matrix. The PCR product was digested with XhoI and KpnI and ligated into digested 2473 YEpGal555 vector.	This study
pB568 YEpGal555 PaExoY_D247A	The D247A mutation was generated by two-step overlap PCR using oligonucleotides cagcgaagcgatgatttttg, caggttgtctaactccttcc, cggtgcaaagcttcccctgagcgag and ctcgctcaggggaagctttgcaccg, and p1593 as a matrix. The PCR product was digested with XhoI and KpnI and ligated into digested 2473 YEpGal555 vector.	This study
pB566 YEpGal555 PaExoY_D257E	The D257E mutation was generated by two-step overlap PCR using oligonucleotides cagcgaagcgatgatttttg, caggttgtctaactccttcc, ggattctatggcgaggaggatatggcc and ggccatatcctcctcgccatagaatcc, and p1593 as a matrix. The PCR product was digested with XhoI and KpnI and ligated into digested 2473 YEpGal555 vector.	This study
pB490 YEpGal555 PaExoY_R233E	The R233E mutation was generated by two-step overlap PCR using oligonucleotides cagcgaagcgatgatttttg, caggttgtctaactccttcc, ggtctcgatgcagaaaggaataccg and cggtattcctttctgcatcgagacc, and p1593 as a matrix. The PCR product was digested with XhoI and KpnI and ligated into digested 2473 YEpGal555 vector.	This study
pB582 YEpGal555 PaExoY_D293R	The D293R mutation was generated by two-step overlap PCR using oligonucleotides cagcgaagcgatgatttttg, caggttgtctaactccttcc, gtttcaccacagccgcgatgcgggcaaccc and gggttgcccgcatcgcggctgtggtgaaac, and p1593 as a matrix. The PCR product was digested with XhoI and KpnI and ligated into digested 2473 YEpGal555 vector.	This study
pB587 YEpGal555 PaExoY_W359S	The W359S mutation was generated by two-step overlap PCR using oligonucleotides cagcgaagcgatgatttttg, caggttgtctaactccttcc, gcccatcccgactcgaacgtgccgc and gcggcacgttcgagtcgggatgggc, and p1593 as a matrix. The PCR product was digested with XhoI and KpnI and ligated into digested 2473 YEpGal555 vector.	This study
pB672 YEpGal555 ChimeraA	The C-terminus of PaExoY was substituted by the homological region of VnExoY by a PCR using oligonucleotides agacctcgagcgtatcgacggtcatcgtcagg and tataggtaccgagtccgttgagcttcgaagattctgtcaaacccagctttttcgccacttcatcccgagcgtctaagtaatcgggccgcagcggcacgttccagtcggg, and p1593 as a matrix. The PCR product was digested with XhoI and KpnI and ligated into digested 2473 YEpGal555 vector.	This study
pB678 YEpGal555 ChimeraB	The central region of PaExoY was substituted by the homological region of VnExoY by the two-step overlap PCR using oligonucleotides cagcgaagcgatgatttttg, caggttgtctaactccttcc, gacttatcacctaagtataaagcgcgttatgacaatcaagctctttacgagaaacaggatggcgcaagtttgggaaacatcactccgcgcacgcggcaac and cgcgctttatacttaggtgataagtcttcgtaggttaccgattctttccactgttcccacgtgagcggttgccttcttgcatcgagaccaccactgccatg. The PCR product was digested with XhoI and KpnI and ligated into digested 2473 YEpGal555 vector.	This study

pB681 YEpGal555 ChimeraE	The C-terminus of PaExoY was substituted by the homological region of VnExoY by a PCR using oligonucleotides agacctcgagcgtatcgacggtcatcgtcagg and tataggtaccgagtccgttgagcttcgaagattctgtcaaacccagctttttcgccacttcatcccgagcgtctaagtaat cgggccgcagcggcacgttccagtcggg, and pB678 as a matrix. The PCR product was digested with XhoI and KpnI and ligated into digested 2473 YEpGal555 vector.	This study
pB756 YEpGal555 ChimeraC	The C-terminus of PaExoY was substituted by the homological region of VnExoY by swaping NcoI/KpnI fragment of pB731 with the similar fragment of p1593.	This study
pB731 YEpGal555 ChimeraD	The chimera gene was synthesized by Twist Bioscience, digested with XhoI and KpnI and ligated into digested 2473 YEpGal555 vector.	This study
pB673 YEpGal555 ChimeraF	The C-terminus of VnExoY was first PCR-amplified using oligonucleotides gccgctgcggcccctgttcactacaaaacgtaagctatc and caggttgtctaactccttcc from the matrix p1648. The remaining part of the toxin was PCR-amplified using oligonucleotides ttgtagtgaacaggggccgcagcggcacgttccag and cagcgaagcgatgatttttg from the plasmid p1593. After the PCR reaction with oligonucleotides cagcgaagcgatgatttttg and caggttgtctaactccttcc, and the products of the previous reactions as matrixes, the complete chimera gene was digested with XhoI and KpnI and ligated into digested 2473 YEpGal555 vector.	This study
pB679 YEpGal555 ChimeraG	The C-terminus of VnExoY was first PCR-amplified using oligonucleotides cggaagagcttttccaattttcagcaggtcgcgattaatgcg and caggttgtctaactccttcc from the matrix p1648. The remaining part of the toxin was PCR-amplified using oligonucleotides cgacctgctgaaaattggaaaagctcttccgatccgccacc and cagcgaagcgatgatttttg from the plasmid p1593. After the PCR reaction with oligonucleotides cagcgaagcgatgatttttg and caggttgtctaactccttcc, and the products of the previous reactions as matrixes, the complete chimera gene was digested with XhoI and KpnI and ligated into digested 2473 YEpGal555 vector.	This study
pB680 YEpGal555 ChimeraH	The C-terminus of VnExoY was first PCR-amplified using oligonucleotides ccttccccggcactttttttgatgatgatggtctgg and caggttgtctaactccttcc from the matrix p1648. The remaining part of the toxin was PCR-amplified using oligonucleotides catcatcaaaaaagtgccggggaaggtagaaggtggccg and cagcgaagcgatgatttttg from the plasmid p1593. After the PCR reaction with oligonucleotides cagcgaagcgatgatttttg and caggttgtctaactccttcc, and the products of the previous reactions as matrixes, the complete chimera gene was digested with XhoI and KpnI and ligated into digested 2473 YEpGal555 vector.	This study
pB682 YEpGal555 ChimeraI	The C-terminus of VnExoY was introduced into pB678 by digestion of pB673 with NdeI and exchanging of the segment with the similar region of NdeI-digested pB678	This study
pB683 YEpGal555 ChimeraJ	The C-terminus of VnExoY was introduced into pB678 by digestion of pB679 with NdeI and exchanging of the segment with the similar region of NdeI-digested pB678	This study
pB684 YEpGal555 ChimeraK	The C-terminus of VnExoY was introduced into pB678 by digestion of pB680 with NdeI and exchanging of the segment with the similar region of NdeI-digested pB678	This study
pB717 YEpGal555 ChimeraL	The central region of PaExoY was substituted by the homological region of VnExoY by the two-step overlap PCR using oligonucleotides cagcgaagcgatgatttttg, caggttgtctaactccttcc, gacctcttcctggtagcgcccacctatggggatttaggtccacaggataaggtgaagcaaccgctcacgtgggaaca gtggaaagaatcggtaacctacgaagacttatcacctaagtat and	This study

	tccaccagttgccgcgtgcgtttcaagcgatcgctgaccatgcccaaacttgcgccatcctgtttctcgtaaagagcttg attgtcataacgcgctttatacttaggtgataagtcttcg, and p1593 as a matrix. The PCR product was digested with XhoI and KpnI and ligated into digested 2473 YEpGal555 vector.	
pB719 YEpGal555 ChimeraM	The C-terminus of PaExoY was substituted by the homological region of VnExoY by a PCR using oligonucleotides agacctcgagcgtatcgacggtcatcgtcagg and tataggtaccgagtccgttgagcttcgaagattctgtcaaacccagctttttcgccacttcatcccgagcgtctaagtaat cgggccgcagcggcacgttccagtcggg, and pB717 as a matrix. The PCR product was digested with XhoI and KpnI and ligated into digested 2473 YEpGal555 vector.	This study
pB729 YEpGal555 ChimeraN	The chimera was generated using two-step overlap PCR. 5'of the chimera was PCR-amplified using oligonucleotides cagcgaagcgatgattttg and cctaaatccccataggtgggcgctaccaggaagaggtcataatc, and matrix p1593. The 3'of the chimera was PCR-amplified using oligonucleotides caggttgtctaactccttcc and cttcctggtagcgcccacctatggggatttaggtccacagg, and matrix p1648. The PCR-fragments of the second PCR step with oligonucleotides caggttgtctaactccttcc and cagcgaagcgatgatttttg, and PCR-fragments from the previous reactions, was digested with XhoI and KpnI and ligated into digested 2473 YEpGal555 vector.	This study
pB730 YEpGal555 ChimeraO	The chimera was generated using two-step overlap PCR. 5'of the chimera was PCR-amplified using oligonucleotides cagcgaagcgatgatttttg and catgcgcctcgatcgagtacatcaccgttaacaagtcataatcc, and matrix p1648. The 3'of the chimera was PCR-amplified using oligonucleotides caggttgtctaactccttcc and gttaacggtgatgtactcgatcgaggcgcatggcagtggtg, and matrix p1593. The PCR-fragments of the second PCR step with oligonucleotides caggttgtctaactccttcc and cagcgaagcgatgatttttg, and PCR-fragments from the previous reactions, was digested with XhoI and KpnI and ligated into digested 2473 YEpGal555 vector.	This study
**Plasmids for protein expression in *E. coli***		
2479 pB386 pET28a MBP-His-ExoY	WT MBP-PaExoY fusion protein with N-terminal His-tag	[11]
pUM522	WT VnExoY with C-terminal His-tag	[12]
pB642 pET28a MBP-PaExoY D247A	*ExoY* gene with the mutation was amplified from pB568 using oligonucleotides tatagagctctggtcgtatcgacggtcatcgtca and tataaagcttcagaccttacgttggaaaaagtc, digested with SacI and HindIII and ligated into digested pB137 vector [2]	This study
pB643 pET28a MBP-PaExoY R257E	*ExoY* gene with the mutation was amplified from pB566 using oligonucleotides tatagagctctggtcgtatcgacggtcatcgtca and tataaagcttcagaccttacgttggaaaaagtc, digested with SacI and HindIII and ligated into digested pB137 vector [2]	This study
pB644 pET28a MBP-PaExoY Y240A F254S Y255S	*ExoY* gene with the mutation was amplified from pB636 using oligonucleotides tatagagctctggtcgtatcgacggtcatcgtca and tataaagcttcagaccttacgttggaaaaagtc, digested with SacI and HindIII and ligated into digested pB137 vector [2]	This study
pB610 pET28a MBP-PaExoY R233E	*ExoY* gene with the mutation was amplified from pB490 using oligonucleotides tatagagctctggtcgtatcgacggtcatcgtca and tataaagcttcagaccttacgttggaaaaagtc, digested with SacI and HindIII and ligated into digested pB137 vector [2]	This study
pB637 pET28a MBP-PaExoY D293R	*ExoY* gene with the mutation was amplified from pB582 using oligonucleotides tatagagctctggtcgtatcgacggtcatcgtca and tataaagcttcagaccttacgttggaaaaagtc, digested with SacI and HindIII and ligated into digested pB137 vector [2]	This study
pB638 pET28a MBP-PaExoY W359S	*ExoY* gene with the mutation was amplified from pB587 using oligonucleotides tatagagctctggtcgtatcgacggtcatcgtca and tataaagcttcagaccttacgttggaaaaagtc, digested with SacI and HindIII and ligated into digested pB137 vector [2]	This study

pB575 pET28a MBP-PaExoY ΔC5	C-terminal deletion was generated by PCR reaction with oligonucleotides tatagagctctggtcgtatcgacggtcatcgtca and tatacaagcttcaaaagtcgagcgcctcctggaag, and 2479 pB386 pET28a MBP-His-ExoY as a matrix. The amplified gene was digested with SacI and HindIII and ligated into digested pB137 vector [2]	This study
pB686 pET28a MBP	SalI and KpnI digesting sites were introduced into pB137 vector [2] using oligonucleotides ctgtcgacggttgataaggtacctaagctagctaaa and agcttttagctagcttaggtaccttatcaaccgtcgacagagct to simplify cloning of genes with N-terminal MBP tag	This study
pB687 pET28a MBP-VnExoY	XhoI KpnI fragment of p1648 was ligated into digested with SalI and KpnI pB686 plasmid to generate a fusion protein of VnExoY and MBP	This study
pB771 MBP-PaExoY ΔC27	XhoI KpnI fragment of pB747 was ligated into digested with SalI and KpnI pB686 to generate a fusion protein of VnExoY with the mutation and MBP	This study
pB693 MBP-VvExoY	VvExoY gene was synthesized by Twist Bioscience, digested with XhoI and KpnI and ligated into SalI/KpnI digested pB686.	This study
pB690 pET28a MBP-Chimera A	XhoI KpnI fragment of pB672 was ligated into digested with SalI and KpnI pB686 to generate a fusion protein of the chimera and MBP	This study
pB691 pET28a MBP-Chimera B	XhoI KpnI fragment of pB678 was ligated into digested with SalI and KpnI pB686 to generate a fusion protein of the chimera and MBP	This study
pB775 pET28a MBP-Chimera C	XhoI KpnI fragment of pB756 was ligated into digested with SalI and KpnI pB686 to generate a fusion protein of the chimera and MBP	This study
pB733 pET28a MBP-Chimera D	XhoI KpnI fragment of pB731 was ligated into digested with SalI and KpnI pB686 to generate a fusion protein of the chimera and MBP	This study
**Plasmids for protein expression in insect cells**		
p2098 pFL_ACTB	First, *actB* gene was amplified with oligonucleotides tatataggatccatggatgatgatatcgccgcgctc and tatataaagcttctagaagcatttgcggtggacgatg from cDNA clone (BioCat), digested with BamHI and HindIII, and inserted into pFL vector. Then, the nucleotide sequence encoding a cleavable linker and thymosin β4 were introduced downstream of actin gene using Gibson cloning.	This study
p2336 pFL_ACTB_C272A	C272A mutation was introduced by the QuikChange method using oligonucleotides cttcctgggcatggagtccgctggcatccacgaaactaccttc and gaaggtagtttcgtggatgccagcggactccatgcccaggaag and p2098 as a matrix.	This study

Supplementary Table 3. Amino acid sequence of PaExoY-VnExoY chimeras

Green and blue colors correspond to PaExoY and VnExoY sequence, respectively.

ID	Plasmid	Amino acid sequence
WT PaExoY, p1593		meqkliseedleridghrqvvsnataqpgpllrpadmqaralqdlfdaqgvgvpvehalrmqavarqtntvfgirpverivttlieegfptkgfsv<br>kgkssnwgpqagficvdqhlskredrdtaeirklnlavakgmdggaytqtdlrisrqrlaelvrnfglvadgvgpvrlltaqgpsgkryefearqe<br>pdglyrisrlgrseavqvlaspacglamtadydlflvapsieahgsggldarrntavrytplgakdplsedgfygredmargnitprtrqlvdalnd<br>clgrgehremfhhsddagnpgshmgdnfpatfylpramehrvgeesvrfdevcvvadrksfsllvecikgngyhftahpdwnvplrpsfqea<br>ldffqrkvnpgtaas*
WT VnExoY, p1648		meqkliseedlegynygqalqeaqldiatmkprqrvtanelqlgddnaitnavtseqeatpnqdgshktyqsrdlvlepiqhpksielgmpevd<br>qsvlaevaerenviigvrpvdeksksliaskmysskglfvkakssdwgpmsgfipvdqsfakasarrdlekfneyaeqsilsgnavsanlylnq<br>vrieelvskyesltpleldvdsgmykttatngdqtipfflnkvtvddkelwqvhylregelapfkvigdpvskqpmtadydlltvmytygdlgp<br>qdkvkqpltweqwkesvtyedlspkykarydnqalyekqdgaslgmvsdrlkelkdvintslgrtdglemvhhgaddanpyavmadnfpa<br>tffvpkhffdddglgegkgsiqtyfnvneqgavviqnpqefsnfqqvainasyraslndkwnsgldsplfttkrklshdyldardevakklgltes<br>sklnglgtaas*
A	pB672	meqkliseedleridghrqvvsnataqpgpllrpadmqaralqdlfdaqgvgvpvehalrmqavarqtntvfgirpverivttlieegfptkgfsv<br>kgkssnwgpqagficvdqhlskredrdtaeirklnlavakgmdggaytqtdlrisrqrlaelvrnfglvadgvgpvrlltaqgpsgkryefearqe<br>pdglyrisrlgrseavqvlaspacglamtadydlflvapsieahgsggldarrntavrytplgakdplsedgfygredmargnitprtrqlvdalnd<br>clgrgehremfhhsddagnpgshmgdnfpatfylpramehrvgeesvrfdevcvvadrksfsllvecikgngyhftahpdwnvplrpdylda<br>rdevakklgltessklnglgtaas*
B	pB678	meqkliseedleridghrqvvsnataqpgpllrpadmqaralqdlfdaqgvgvpvehalrmqavarqtntvfgirpverivttlieegfptkgfsv<br>kgkssnwgpqagficvdqhlskredrdtaeirklnlavakgmdggaytqtdlrisrqrlaelvrnfglvadgvgpvrlltaqgpsgkryefearqe<br>pdglyrisrlgrseavqvlaspacglamtadydlflvapsieahgsggldarrqpltweqwkesvtyedlspkykarydnqalyekqdgaslgni<br>tprtrqlvdalndclgrgehremfhhsddagnpgshmgdnfpatfylpramehrvgeesvrfdevcvvadrksfsllvecikgngyhftahpd<br>wnvplrpsfqealdffqrkvnpgtaas*
C	pB756	meqkliseedleridghrqvvsnataqpgpllrpadmqaralqdlfdaqgvgvpvehalrmqavarqtntvfgirpverivttlieegfptkgfsv<br>kgkssnwgpqagficvdqhlskredrdtaeirklnlavakgmdggaytqtdlrisrqrlaelvrnfglvadgvgpvrlltaqgpsgkryefearqe<br>pdglyrisrlgrseavqvlaspacglamtadydlflvaptygdlgpqdkvkqpltweqwkesvtyedlspkykarydnqalyekqdgaslgm<br>vsdrlkelkdvintslgrtdglemvhhgaddanpgshmgdnfpatfylpramehrvgeesvrfdevcvvadrksfsllvecikgngyhftahp<br>dwnvplrpsfqealdffqrkvnpgtaas*
D	pB731	meqkliseedleridghrqvvsnataqpgpllrpadmqaralqdlfdaqgvgvpvehalrmqavarqtntvfgirpverivttlieegfptkgfsv<br>kgkssnwgpqagficvdqhlskredrdtaeirklnlavakgmdggaytqtdlrisrqrlaelvrnfglvadgvgpvrlltaqgpsgkryefearqe<br>pdglyrisrlgrseavqvlaspacglamtadydlflvaptygdlgpqdkvkqpltweqwkesvtyedlspkykarydnqalyekqdgaslgm<br>vsdrlkelkdvintslgrtdglemvhhgaddanpgshmgdnfpatfylpramehrvgeesvrfdevcvvadrksfsllvecikgngyhftahp<br>dwnvplrpdyldardevakklgltessklnglgtaas*
E	pB681	meqkliseedleridghrqvvsnataqpgpllrpadmqaralqdlfdaqgvgvpvehalrmqavarqtntvfgirpverivttlieegfptkgfsv<br>kgkssnwgpqagficvdqhlskredrdtaeirklnlavakgmdggaytqtdlrisrqrlaelvrnfglvadgvgpvrlltaqgpsgkryefearqe<br>pdglyrisrlgrseavqvlaspacglamtadydlflvapsieahgsggldarrqpltweqwkesvtyedlspkykarydnqalyekqdgaslgni<br>tprtrqlvdalndclgrgehremfhhsddagnpgshmgdnfpatfylpramehrvgeesvrfdevcvvadrksfsllvecikgngyhftahpd<br>wnvplrpdyldardevakklgltessklnglgtaas*
F	pB673	meqkliseedleridghrqvvsnataqpgpllrpadmqaralqdlfdaqgvgvpvehalrmqavarqtntvfgirpverivttlieegfptkgfsv<br>kgkssnwgpqagficvdqhlskredrdtaeirklnlavakgmdggaytqtdlrisrqrlaelvrnfglvadgvgpvrlltaqgpsgkryefearqe<br>pdglyrisrlgrseavqvlaspacglamtadydlflvapsieahgsggldarrntavrytplgakdplsedgfygredmargnitprtrqlvdalnd<br>clgrgehremfhhsddagnpgshmgdnfpatfylpramehrvgeesvrfdevcvvadrksfsllvecikgngyhftahpdwnvplrplfttkr<br>klshdyldardevakklgltessklnglgtaas*
G	pB679	meqkliseedleridghrqvvsnataqpgpllrpadmqaralqdlfdaqgvgvpvehalrmqavarqtntvfgirpverivttlieegfptkgfsv<br>kgkssnwgpqagficvdqhlskredrdtaeirklnlavakgmdggaytqtdlrisrqrlaelvrnfglvadgvgpvrlltaqgpsgkryefearqe<br>pdglyrisrlgrseavqvlaspacglamtadydlflvapsieahgsggldarrntavrytplgakdplsedgfygredmargnitprtrqlvdalnd<br>clgrgehremfhhsddagnpgshmgdnfpatfylpramehrvgeesvrfdevcvvadrksfsnfqqvainasyraslndkwnsgldsplfttk<br>rklshdyldardevakklgltessklnglgtaas*
H	pB680	meqkliseedleridghrqvvsnataqpgpllrpadmqaralqdlfdaqgvgvpvehalrmqavarqtntvfgirpverivttlieegfptkgfsv<br>kgkssnwgpqagficvdqhlskredrdtaeirklnlavakgmdggaytqtdlrisrqrlaelvrnfglvadgvgpvrlltaqgpsgkryefearqe<br>pdglyrisrlgrseavqvlaspacglamtadydlflvapsieahgsggldarrntavrytplgakdplsedgfygredmargnitprtrqlvdalnd

		clgrgehremfhhsddagnpgshmgdnfpatfylprhffdddglgegkgsiqtyfnvneqgavviqnpqefsnfqqvainasyraslndkwnsgldsplfttkrklshdyldardevakklgltessklnglgtaas*
I	pB682	meqkliseedleridghrqvvsnataqpgpllrpadmqaralqdlfdaqgvgvpvehalrmqavarqtntvfgirpverivttlieegfptkgfsvkgkssnwgpqagficvdqhlskredrdtaeirklnlavakgmdggaytqtdlrisrqrlaelvrnfglvadgvgpvrlltaqgpsgkryefearqepdglyrisrlgrseavqvlaspacglamtadydlflvapsieahgsggldarrqpltweqwkesvtyedlspkykarydnqalyekqdgaslgnitprtrqlvdalndclgrgehremfhhsddagnpgshmgdnfpatfylpramehrvgeesvrfdevcvvadrksfsllvecikgngyhftahpdwnvplrplfttkrklshdyldardevakklgltessklnglgtaas*
J	pB683	meqkliseedleridghrqvvsnataqpgpllrpadmqaralqdlfdaqgvgvpvehalrmqavarqtntvfgirpverivttlieegfptkgfsvkgkssnwgpqagficvdqhlskredrdtaeirklnlavakgmdggaytqtdlrisrqrlaelvrnfglvadgvgpvrlltaqgpsgkryefearqepdglyrisrlgrseavqvlaspacglamtadydlflvapsieahgsggldarrqpltweqwkesvtyedlspkykarydnqalyekqdgaslgnitprtrqlvdalndclgrgehremfhhsddagnpgshmgdnfpatfylpramehrvgeesvrfdevcvvadrksfsnfqqvainasyraslndkwnsgldsplfttkrklshdyldardevakklgltessklnglgtaas*
K	pB684	meqkliseedleridghrqvvsnataqpgpllrpadmqaralqdlfdaqgvgvpvehalrmqavarqtntvfgirpverivttlieegfptkgfsvkgkssnwgpqagficvdqhlskredrdtaeirklnlavakgmdggaytqtdlrisrqrlaelvrnfglvadgvgpvrlltaqgpsgkryefearqepdglyrisrlgrseavqvlaspacglamtadydlflvapsieahgsggldarrqpltweqwkesvtyedlspkykarydnqalyekqdgaslgnitprtrqlvdalndclgrgehremfhhsddagnpgshmgdnfpatfylprhffdddglgegkgsiqtyfnvneqgavviqnpqefsnfqqvainasyraslndkwnsgldsplfttkrklshdyldardevakklgltessklnglgtaas*
L	pB717	meqkliseedleridghrqvvsnataqpgpllrpadmqaralqdlfdaqgvgvpvehalrmqavarqtntvfgirpverivttlieegfptkgfsvkgkssnwgpqagficvdqhlskredrdtaeirklnlavakgmdggaytqtdlrisrqrlaelvrnfglvadgvgpvrlltaqgpsgkryefearqepdglyrisrlgrseavqvlaspacglamtadydlflvaptygdlgpqdkvkqpltweqwkesvtyedlspkykarydnqalyekqdgaslgmvsdrlkrtrqlvdalndclgrgehremfhhsddagnpgshmgdnfpatfylpramehrvgeesvrfdevcvvadrksfsllvecikgngyhftahpdwnvplrpsfqealdffqrkvnpgtaas*
M	pB719	meqkliseedleridghrqvvsnataqpgpllrpadmqaralqdlfdaqgvgvpvehalrmqavarqtntvfgirpverivttlieegfptkgfsvkgkssnwgpqagficvdqhlskredrdtaeirklnlavakgmdggaytqtdlrisrqrlaelvrnfglvadgvgpvrlltaqgpsgkryefearqepdglyrisrlgrseavqvlaspacglamtadydlflvaptygdlgpqdkvkqpltweqwkesvtyedlspkykarydnqalyekqdgaslgmvsdrlkrtrqlvdalndclgrgehremfhhsddagnpgshmgdnfpatfylpramehrvgeesvrfdevcvvadrksfsllvecikgngyhftahpdwnvplrpdyldardevakklgltessklnglgtaas*
N	pB729	meqkliseedleridghrqvvsnataqpgpllrpadmqaralqdlfdaqgvgvpvehalrmqavarqtntvfgirpverivttlieegfptkgfsvkgkssnwgpqagficvdqhlskredrdtaeirklnlavakgmdggaytqtdlrisrqrlaelvrnfglvadgvgpvrlltaqgpsgkryefearqepdglyrisrlgrseavqvlaspacglamtadydlflvaptygdlgpqdkvkqpltweqwkesvtyedlspkykarydnqalyekqdgaslgmvsdrlkelkdvintslgrtdglemvhhgaddanpyavmadnfpatffvpkhffdddglgegkgsiqtyfnvneqgavviqnpqefsnfqqvainasyraslndkwnsgldsplfttkrklshdyldardevakklgltessklnglgtaas*
O	pB730	meqkliseedlegynygqalqeaqldiatmkprqrvtanelqlgddnaitnavtseqeatpnqdgshktyqsrdlvlepiqhpksielgmpevdqsvlaevaerenviigvrpvdeksksliaskmysskglfvkakssdwgpmsgfipvdqsfakasarrdlekfneyaeqsilsgnavsanlylnqvrieelvskyesltpleldvdsgmykttatngdqtipfflnkvtvddkelwqvhylregelapfkvigdpvskqpmtadydlltvmysieahgsggldarrntavrytplgakdplsedgfygredmargnitprtrqlvdalndclgrgehremfhhsddagnpgshmgdnfpatfylpramehrvgeesvrfdevcvvadrksfsllvecikgngyhftahpdwnvplrpsfqealdffqrkvnp*

Supplementary Table 4. Overview of the protocol used for the MD simulations.

Step	Time	Positional restraints	Restraints force constant	Temperature control	Pressure control	Additional restraints
			**Free PaExoY**			
Heating	0.072 ns	All heavy atom excluding water and ions	5 kcal/mol/A^2	Langevin, t=50 - 300 K Damping = 1 ps^{-1}	None	None
Equilibration 1	0.928 ns	All heavy atom excluding water and ions	5 kcal/mol/A^2	Langevin, t=300 K Damping = 1 ps^{-1}	Nosé-Hoover Langevin, p= 1 atm Period = 100 fs Decay = 50 fs	None
Equilibration 2	2.5 ns	All heavy atom excluding water and ions	2 kcal/mol/A^2	Langevin, t=300 K Damping = 1 ps^{-1}	Nosé-Hoover Langevin, p= 1 atm Period = 50 fs Decay = 25 fs	None
Equilibration 3	2.5 ns	Protein backbone	2 kcal/mol/A^2	Langevin, t=300 K Damping = 1 ps^{-1}	Nosé-Hoover Langevin, p= 1 atm Period = 50 fs Decay = 25 fs	None
Equilibration 4	5 ns	Protein backbone except aa. 107 – 116, 317 - 326	2 kcal/mol/A^2	Langevin; 300 K Damping = 1 ps -1	Nosé-Hoover Langevin, p= 1 atm Period = 50 fs Decay = 25 fs	None
Equilibration 5	5 ns	None	-	Langevin, t=300 K Damping = 1 ps^{-1}	Nosé-Hoover Langevin, p= 1 atm Period = 50 fs Decay = 25 fs	None
Production	400 ns	None	-	Langevin, t=300 K Damping = 1 ps^{-1}	Nosé-Hoover Langevin, p= 1 atm Period = 50 fs Decay = 25 fs	None
			**F-actin PaExoY**			
Heating	0.072 ns	All heavy atom excluding water and ions	5 kcal/mol/A^2	Langevin, t=50 - 300 K Damping = 1 ps^{-1}	None	Mg coordination d = 2Å K = 50 kcal/mol/A^2
Equilibration 1	0.928 ns	All heavy atom excluding water and ions	5 kcal/mol/A^2	Langevin, t=300 K Damping = 1 ps^{-1}	Nosé-Hoover Langevin, p= 1 atm Period = 100 fs Decay = 50 fs	Mg coordination d = 2Å K = 50 kcal/mol/A^2
Equilibration 2	2.5 ns	All heavy atom excluding water and ions	2 kcal/mol/A^2	Langevin, t=300 K Damping = 1 ps^{-1}	Nosé-Hoover Langevin, p= 1 atm Period = 50 fs Decay = 25 fs	Mg coordination d = 2Å K = 50 kcal/mol/A^2
Equilibration 3	2.5 ns	Protein backbone	2 kcal/mol/A^2	Langevin, t=300 K Damping = 1 ps^{-1}	Nosé-Hoover Langevin, p= 1 atm Period = 50 fs Decay = 25 fs	Mg coordination d = 2Å K = 50 kcal/mol/A^2
Equilibration 4	5 ns	Protein backbone except: actin aa. 1 – 4; ExoY aa. 107 – 116, aa. 317 - 326	2 kcal / mol A2	Langevin, t=300 K Damping = 1 ps^{-1}	Nosé-Hoover Langevin, p= 1 atm Period = 50 fs Decay = 25 fs	Mg coordination d = 2Å K = 50 kcal/mol/A^2
Equilibration 5	5 ns	None	-	Langevin, t=300 K Damping = 1 ps^{-1}	Nosé-Hoover Langevin, p= 1 atm Period = 50 fs Decay = 25 fs	Mg coordination d = 2Å K = 50 kcal/mol/A^2; Orientation restraint (orientation colvar) Restraint on every 5th CA of the last two protomers of each of actin's end K = 1000
Production	400 ns	None	-	Langevin, t=300 K Damping = 1 ps^{-1}	Nosé-Hoover Langevin, p= 1 atm Period = 50 fs Decay = 25 fs	Mg coordination d = 2Å K = 50 kcal/mol/A^2 Orientation restraint (orientation colvar) Restraint on every 5th CA of the last two protomers of each of actin's end K = 400

References

1. Khanppnavar, B. & Datta, S. Crystal structure and substrate specificity of ExoY, a unique T3SS mediated secreted nucleotidyl cyclase toxin from Pseudomonas aeruginosa. *Biochim Biophys Acta Gen Subj* **1862,** 2090–2103 (2018).
2. Belyy, A. *et al.* The extreme C terminus of the Pseudomonas aeruginosa effector ExoY is crucial for binding to its eukaryotic activator, F-actin. *J Biol Chem* **293,** 19785–19796 (2018).
3. Guo, Q. *et al.* Structural basis for the interaction of Bordetella pertussis adenylyl cyclase toxin with calmodulin. *EMBO J* **24,** 3190–3201 (2005).
4. Shen, Y., Zhukovskaya, N. L., Guo, Q., Florián, J. & Tang, W.-J. Calcium-independent calmodulin binding and two-metal-ion catalytic mechanism of anthrax edema factor. *EMBO J* **24,** 929–941 (2005).
5. Sanchez-Garcia, R. *et al.* DeepEMhancer: a deep learning solution for cryo-EM volume post-processing. *bioRxiv* 2020.06.12.148296 (2020).
6. Tan, Y. Z. *et al.* Addressing preferred specimen orientation in single-particle cryo-EM through tilting. *Nat Methods* **14,** 793–796 (2017).
7. Notredame, C., Higgins, D. G. & Heringa, J. T-Coffee: A novel method for fast and accurate multiple sequence alignment. *J Mol Biol* **302,** 205–217 (2000).
8. Peisker, K. *et al.* Ribosome-associated complex binds to ribosomes in close proximity of Rpl31 at the exit of the polypeptide tunnel in yeast. *Mol Biol Cell* **19,** 5279–5288 (2008).
9. Raoux-Barbot, D. *et al.* Differential regulation of actin-activated nucleotidyl cyclase virulence factors by filamentous and globular actin. *PLoS One* **13,** e0206133 (2018).
10. Belyy, A. *et al.* Roles of Asp179 and Glu270 in ADP-Ribosylation of Actin by Clostridium perfringens Iota Toxin. *PLoS One* **10,** e0145708 (2015).
11. Belyy, A., Merino, F., Sitsel, O. & Raunser, S. Structure of the Lifeact–F-actin complex. *PLOS Biology* **18,** e3000925 (2020).
12. Belyy, A. *et al.* Actin activates Pseudomonas aeruginosa ExoY nucleotidyl cyclase toxin and ExoY-like effector domains from MARTX toxins. *Nat Commun* **7,** 13582 (2016).
