# Supplementary material for: Mechanism of actin-dependent activation of nucleotidyl cyclase toxins from bacterial human pathogens
Source: Nat Commun. 2021 Nov 16;12:6628. doi: 10.1038/s41467-021-26889-2 (PMC8595890; doi:10.1038/s41467-021-26889-2)
Supplement: Supplementary file 3 — Description of Additional Supplementary Files [file 41467_2021_26889_MOESM3_ESM.pdf]

Description of Additional Supplementary Files

Supplementary Movie 1. Molecular dynamics simulations of free PaExoY and in complex with F-actin. For guidance, the starting structure of PaExoY is shown as translucent. For the F-actin complexes, we show only the initial structure of the filament as surface.

Supplementary Movie 2. Mechanism of activation of PaExoY upon binding to F-actin.
