# Supplementary material for: Mechanism of actin-dependent activation of nucleotidyl cyclase toxins from bacterial human pathogens
Source: Nat Commun. 2021 Nov 16;12:6628. doi: 10.1038/s41467-021-26889-2 (PMC8595890; doi:10.1038/s41467-021-26889-2)
Supplement: Supplementary file 6 — Reporting summary [file 41467_2021_26889_MOESM6_ESM.pdf]

nature portfolio

Corresponding author(s): Stefan Raunser

Last updated by author(s): October 18, 2021

Reporting Summary

Nature Portfolio wishes to improve the reproducibility of the work that we publish. This form provides structure for consistency and transparency in reporting. For further information on Nature Portfolio policies, see our Editorial Policies and the Editorial Policy Checklist.

Statistics

For all statistical analyses, confirm that the following items are present in the figure legend, table legend, main text, or Methods section.

n/a	Confirmed	
☐	☒	The exact sample size (n) for each experimental group/condition, given as a discrete number and unit of measurement
☐	☒	A statement on whether measurements were taken from distinct samples or whether the same sample was measured repeatedly
☒	☐	The statistical test(s) used AND whether they are one- or two-sided<br>*Only common tests should be described solely by name; describe more complex techniques in the Methods section.*
☒	☐	A description of all covariates tested
☒	☐	A description of any assumptions or corrections, such as tests of normality and adjustment for multiple comparisons
☐	☒	A full description of the statistical parameters including central tendency (e.g. means) or other basic estimates (e.g. regression coefficient) AND variation (e.g. standard deviation) or associated estimates of uncertainty (e.g. confidence intervals)
☒	☐	For null hypothesis testing, the test statistic (e.g. F, t, r) with confidence intervals, effect sizes, degrees of freedom and P value noted<br>*Give P values as exact values whenever suitable.*
☒	☐	For Bayesian analysis, information on the choice of priors and Markov chain Monte Carlo settings
☒	☐	For hierarchical and complex designs, identification of the appropriate level for tests and full reporting of outcomes
☒	☐	Estimates of effect sizes (e.g. Cohen's d, Pearson's r), indicating how they were calculated

Our web collection on statistics for biologists contains articles on many of the points above.

Software and code

Policy information about availability of computer code

Data collection	- Cryo-EM data: EPU version 2.7 and 2.8 (Thermo Fisher Scientific)<br>- SDS-PAGE and western blots: ImageLab version 5.2.1
Data analysis	- Cryo-EM data: crYOLO versions 1.5, 1.6, 1.8; CTFFIND versions 4.1.10, 4.1.13; MotionCor2 versions 1.1.0, 1.2.6, 1.3; TranSPHIRE versions 1.4.28, 1.5.13; SPHIRE versions 1.3, 1.4; Relion versions 3, 3.1; iMODFIT Chimera plugin version 1.2; DeepEMhancer version 1.0; Rosetta version 3; PHENIX version 1.17; ISOLDE version 1.0B4; UCSF Chimera version 1.14.<br>- MD simulations and analysis: NAMD version 2.14, VMD 1.9.4alpha and Carma 2.01<br>- Kd calculations: Prism versions 8 and 9

For manuscripts utilizing custom algorithms or software that are central to the research but not yet described in published literature, software must be made available to editors and reviewers. We strongly encourage code deposition in a community repository (e.g. GitHub). See the Nature Portfolio guidelines for submitting code & software for further information.

Data

Policy information about availability of data

All manuscripts must include a data availability statement. This statement should provide the following information, where applicable:
- Accession codes, unique identifiers, or web links for publicly available datasets
- A description of any restrictions on data availability
- For clinical datasets or third party data, please ensure that the statement adheres to our policy

The coordinates for the cryo-EM structures of PaExoY-F-actin, VvExoY-G-actin-profilin and VnExoY-G-actin have been deposited in the Electron Microscopy Data Bank under accession numbers EMD-13158, 13159, 13160. The corresponding molecular models for the PaExoY-F-actin, VvExoY-G-actin-profilin complexes have

been deposited at the wwPDB with accession codes PDB 7P1G, 7P1H. The raw data generated during the current study are available from the corresponding author on reasonable request. Individual data points from the graphs at Fig. 4B, 4C, 4D, Supplementary Fig. 2E, 2G, 3B, 3D, 3G and 5 are available in Source Data. Uncropped gels and Western blots from Supplementary Fig. 2D, 2F, 3E, 3F and 8C can be found in Supplementary Fig. 10.

Field-specific reporting

Please select the one below that is the best fit for your research. If you are not sure, read the appropriate sections before making your selection.

[x] Life sciences [ ] Behavioural & social sciences [ ] Ecological, evolutionary & environmental sciences

For a reference copy of the document with all sections, see nature.com/documents/nr-reporting-summary-flat.pdf

Life sciences study design

All studies must disclose on these points even when the disclosure is negative.

Sample size	n=3; Sample size was chosen based on the previous publications (Belyy et al., JBC 2018, Belyy et al., Nat Commun 2016)
Data exclusions	No data were excluded.
Replication	Measurements of nucleotidyl cyclase activity and cosedimentation assays were performed independently three times.
Randomization	No randomization was necessary as all data, which passed quiality control, were used for analysis.
Blinding	Investigators were not blinded during data acquisition or analysis.

Reporting for specific materials, systems and methods

We require information from authors about some types of materials, experimental systems and methods used in many studies. Here, indicate whether each material, system or method listed is relevant to your study. If you are not sure if a list item applies to your research, read the appropriate section before selecting a response.

Materials & experimental systems

n/a	Involved in the study	
[ ]	[x]	Antibodies
[ ]	[x]	Eukaryotic cell lines
[x]	[ ]	Palaeontology and archaeology
[x]	[ ]	Animals and other organisms
[x]	[ ]	Human research participants
[x]	[ ]	Clinical data
[x]	[ ]	Dual use research of concern

Methods

n/a	Involved in the study	
[x]	[ ]	ChIP-seq
[x]	[ ]	Flow cytometry
[x]	[ ]	MRI-based neuroimaging

Antibodies

Antibodies used	myc-tag (9B11, reference #2276) mouse mAb Cell signaling technology, lot 24, reference 02/2019, dilution 1:10000; RPS9 polyclonal rabbit antibody (used at dilution 1:10000), produced by Eurogentec, is a present of Prof. S. Rospert (University of Freiburg)
Validation	"Myc-Tag (9B11) Mouse mAb detects recombinant proteins containing the Myc epitope tag. The antibody recognizes the Myc-tag fused to either the amino or carboxy terminus of targeted proteins in transfected cells. The antibody may cross-react with c-myc protein. The antibody may weakly cross-react with a protein of unknown origin ~90kDa." https://www.cellsignal.de/products/primary-antibodies/myc-tag-9b11-mouse-mab/2276<br><br>Anti-RPS9 antibody was validated by the side-by-side western blot analysis of the WT yeast strain and a strain with tagged RPS9 protein (Raue et al., JBC 2007; Zhang et al., Nat Commun 2021)

Eukaryotic cell lines

Policy information about cell lines

Cell line source(s)	BTI-Tnao38, species of origin - Trichoplusia ni. Provider - Boyce Thompson Institute for Plant Research, Inc., 533 Tower Road, Ithaca, NY 14853
Authentication	The BTI-Tnao38 cell line was not authenticated.
Mycoplasma contamination	The cells were not tested for mycoplasma contamination.

Commonly misidentified lines
(See ICLAC register)

Name any commonly misidentified cell lines used in the study and provide a rationale for their use.
